# Population growth of microcrustaceans in water from habitats with differing salinities

**DOI:** 10.7717/peerj.12378

**Published:** 2021-11-09

**Authors:** Christopher J. Breen, Abigail E. Cahill

**Affiliations:** Biology Department, Albion College, Albion, Michigan, USA

**Keywords:** Salinity, Copepods, Ostracods, Invasive species, Species interactions

## Abstract

Inland salt marshes are a rare habitat in North America. Little is known about the invertebrates in these habitats and their ability to cope with the brackish conditions of the marsh. We studied the population growth of ostracods found in an inland salt marsh (Maple River salt marsh) and of copepods found in the wetland habitat immediately adjacent to the freshwater Kalamazoo River. By studying these species in water from both habitats, we aimed to find out if they performed differently in the two habitats. We also tested *Daphnia pulex* in water from the two habitats due to the history of *Daphnia* spp. as model organisms. We found that copepods performed better in water taken from the Maple River salt marsh, and the ostracods and *D. pulex* performed equally well in either water. This was unexpected, since ostracods are found in the salt marsh and copepods in the freshwater area. As a second experiment, we tested the invertebrates in pairwise interactions. In water from the Kalamazoo River, ostracods outperformed the other two species, but there was no difference between *D. pulex* and copepods. No species outperformed the other in salt marsh water. Our results show no local adaptation to salinity, suggesting that ostracods and copepods may be limited in their respective distributions by dispersal limitation or habitat suitability.

## Introduction

Biotic and abiotic factors are both important for how well organisms do in a certain environment, helping to shape the population’s demographics through stressors (Arnott & Vanni, 1993; Conte et al., 2007). The organisms of Michigan’s inland salt marshes are no exception to this rule. These salt marshes are a rare and patchy environment that exists due to salt deposits left behind when the current Great Lakes region of North America was covered by ocean during the Silurian period. Pleistocene glaciation scraped away many layers of sediment and allowed water to access these salt deposits, creating salt seeps (Albert, 2010; Eallonardo & Leopold, 2013). In this paper, we use “inland salt marsh” to refer exclusively to marshes of this origin and do not include other inland bodies of saline water (*e.g.*, hypersaline lakes).

These inland salt marshes are very different from coastal salt marshes. One of the most drastic differences between inland marshes and coastal salt marshes is that coastal salt marshes are connected to the ocean, which controls their water level. Conversely, inland marshes mostly rely on rainfall and groundwater seepage to keep the marsh wet. This means that it is possible for inland marshes to dry up if there is no rain, creating a different environment that may not be suitable for some of its aquatic inhabitants (Bai et al., 2017).

Since inland salt marshes are extremely rare, very little is known about their ecology, including phenomena such as the complex relationship between salinity and its effects on the species living there. Moreover, bodies of freshwater in the same region are becoming increasingly saline due to human disturbance. In the Great Lakes region as well as other parts of midwestern and northeastern North America, the number one way to clear snow and ice is road salt (Bolen, 2014). This road salt can end up in nearby waterbodies, which are thus at risk of long-term salinization and corresponding ecological impacts (Dugan et al., 2017; Hintz & Relyea, 2019; Kaushal et al., 2021). Knowing how varying salt concentrations affect certain species may help us better understand the impact of using road salt.

Generally, species that have lived in freshwater do not transition well when introduced to water that has a higher salinity (Hart et al., 1991; Wheeler et al., 2009). There can be many adverse physiological and demographic effects of salinity changes, the most extreme of which is mortality (Hassell, Kefford & Nugegoda, 2006). Even small changes in salinity can cause changes in the structure and composition of freshwater communities (Hintz et al., 2017; Venâncio et al., 2018). While many freshwater species struggle to survive changing salinities, some vertebrates and invertebrates are adapted to survive in brackish water with changing salinity (Goldstein, Oppelt & Maren, 1968; Ali et al., 2005; Svetlichny & Hubareva, 2014).

Given the potential for the salinity levels in inland salt marshes to pose abiotic challenges to species that live there, we used this habitat in a common garden laboratory experiment that tested population growth of multiple invertebrate species in response to differing salinities. The two sites we will be focusing on are the Maple River salt marsh and the wetland habitat immediately adjacent to the Kalamazoo River in Albion, Michigan.

The Maple River salt marsh (MRSM) is an inland salt marsh found in Fowler, Michigan (USA). The marsh itself is not connected to the Maple River and is fed by groundwater and rainfall, meaning that the water level varies during the year (Lincoln et al., 2020). Although the marsh covers an area of 6.5 acres, the area strongly impacted by the salt seep is much smaller, approximately 10 m in diameter (Lincoln et al., 2020). Our previous work has shown that salinity can range up to nearly five ppt at the seep itself but declines rapidly from that point and varies through the year (Cahill et al., 2021). The vegetation of the MRSM has been described (Albert, 2010; Eallonardo & Leopold, 2013; Lincoln et al., 2020), but the invertebrate community remains relatively unknown (Albert, 2010). In addition to the MRSM, we sampled a wetland habitat as an interesting and logistically feasible reference site immediately adjacent to the Kalamazoo River in Albion, Michigan, which flows through the Whitehouse Nature Center (WNC) on Albion College’s campus.

The MRSM is home to an unidentified species of ostracod (a small crustacean commonly known as a seed or clam shrimp). Ostracods are found in a broad range of aquatic environments and can live in the ocean as well as lakes and ponds (Salel, Bruneton & Lefèvre, 2016). Although the group as a whole has a broad salinity tolerance, each species has a different preferred environment, as well as a different tolerance to stressful conditions (Herrewege & David, 1997; Yamori et al., 2010). Ostracods have a history of being used as environmental tracers; species of ostracod have shown a change in the carapace shape with environmental factors such as pH, salinity, and temperature (Ruiz et al., 2013). Barcoding of the ostracods from the MRSM with the cytochrome oxidase I (COI) gene yielded a 95% match to *Heterocypris salina* (Cahill et al., 2021), but it is not clear if the species is in fact *H. salina* or a different species. In other studies, *H. salina* had the ability to survive in water with a salinity ranging from five ppt to around 28 ppt (Ruiz et al., 2013); Ganning (1971) found a preference for six ppt in a Baltic population of *H. salina*. Since we are not certain of the species-level identification of the MRSM ostracods, for the rest of this study we will refer to these ostracods as *Heterocypris* sp. A. Although in-depth studies are warranted to confirm the distribution range of the species in the region, so far we have not detected *Heterocypris* sp. A in the Kalamazoo River.

The other species that we used in this experiment lives in the relatively still water of the collapsed riverbanks of the Kalamazoo River: a species of cyclopoid copepod. Copepods are found in many aquatic habitats, including but not limited to vernal pools, tide pools, and the ocean (Coull et al., 1979). Many species of copepods live in salty conditions, but the ones we worked with are accustomed to a freshwater environment. A thorough sampling of the marsh in 2018 did not yield any traces of copepods at the Maple River salt marsh, despite using with both morphological and molecular methods (Cahill et al., 2021). Other species of freshwater copepod have been shown to react adversely when temperature and salinity change in their environment (*e.g., Boeckella hamata*; Hall & Burns, 2001). We expected our species of copepod to react in a similar manner to the ones mentioned in the experiment by Hall and Burns–that is, to show lower population growth in response to changing salinity. We have made two attempts to barcode these copepods using COI. In the year prior to our experiment, the copepods that we sequenced had a 78% match to *Diacyclops incolotaenia*. Our second attempt to barcode the copepods, during our experiment, was only slightly more successful (91% match to an unidentified cyclopoid copepod). *Diacyclops* is a widespread and common genus of freshwater cyclopoid copepods (Stoch, 2001), including in the Great Lakes region (Stemberger, 1985). Since the barcoding did not yield a clear species-level identification, when referring to the species of copepod we will refer to them as *Diacyclops* sp. A.

Although we were unable to identify either the ostracods or the copepods to the species level, this was not the main focus of our work. Instead, we focused on the response of these species to their habitats; we were more interested in the ability of these species to survive in unfamiliar habitats, independent of their identity.

*Daphnia* species have a long history of being used as model organisms in part due to their sensitivity to stressful conditions, including salinity stress (*e.g.*, in *D. dentifera*, Searle et al., 2016; *D. longispina*, Gonçalves et al., 2007). There is variation in salinity tolerance in field-collected populations of the freshwater *D. pulex* (Weider & Hebert, 1987; Liu & Steiner, 2017). While *D. pulex* was not found at either site, we used them as a reference for our experiment. Having a well-studied reference species allowed us to better understand the reactions of the other two species, about which we did not have strong *a priori* expectations.

Since no copepods have been found at the MRSM, and *Heterocypris* sp. A. has not been found in the Kalamazoo River, we wanted to know if these animals were adapted to their local salinity conditions. We tested this by placing each invertebrate species in water from its own environment and water from the other environment and measured population growth to see if the individuals would be able to survive in this new water. *Daphnia pulex* were placed in both sets of water as well. Our hypothesis was that each species would have higher population growth in the water that it originated from, and that *D. pulex* would have higher population growth in the water taken from the Kalamazoo River since it is a freshwater environment.

Following the first experiment, we chose to see if the outcome for a particular species within a habitat was changed by interactions with other species. We therefore set up a second experiment with species placed in pairwise trials in the two different water types (salty and fresh). Our hypothesis was that the species that was in its home environment would perform better than the other (*e.g., Heterocypris* sp. A would outperform *Diacyclops* sp. A in water taken from the MRSM). When one of the invertebrates was placed with *D. pulex* no matter the environment, we predicted that the *D. pulex* would be outperformed because *D. pulex* are not native to either the MRSM or the Kalamazoo River.

## Materials & methods

We collected invertebrates from two locations in late May and early June of 2019. The first was the Kalamazoo River (42.2452 N, 84.7290 W), located in the Whitehouse Nature Center at Albion College (hereafter WNC) and the second was the inland salt marsh in the Maple River State Game Area (43.1195 N, 84.6532 W), located in Fowler, Michigan (hereafter MRSM). Sampling at the MRSM was conducted with approval of the Michigan Department of Natural Resources (permit MR-01-19). Sampling at the WNC was conducted in small pools adjacent to the river because our target species (*Diacyclops* sp. A) does not live in the moving water; these pools had standing water in them but are connected to the main channel when water levels are high (they were not connected at the time of sampling). The area surrounding both sample sites was dominated by cattails (*Typha* sp.), but where the samples were taken from had no vegetation. At both sites, we collected sediment by scooping it into plastic containers and collected water in carboys. Although both ostracods and copepods are groups known to contain cryptic species (*e.g.*, Monchenko, 2000), our samples were collected in a very small area (~1 m square), reducing the chances that we were accidentally sampling multiple species. Sediment samples were kept in a fridge at 5 °C until use (1–2 days for both sampling locations). Water samples were filtered using 5 µm bag filters before use, and stored at room temperature for the duration of the experiment. Although a 5 µm filter removed large organisms and particulates and some bacteria, we expect that smaller bacteria and other microbes were retained in the cultures. Salinity of the water from the two sites was measured with a YSI probe (model Pro2030). The salinity of the water from the WNC was 0.3 ppt and water from the MRSM was 2.5 ppt. All studies were conducted in compliance with the US National Research Council’s Guide for the Care and Use of Laboratory Animals.

### Experiment 1

**Figure 1:**
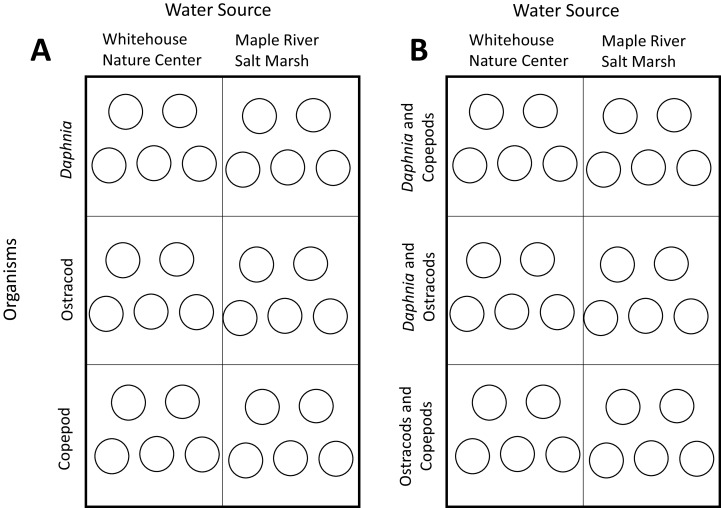
Experimental layouts. (A) Layout of experiment one. Circles represent a single glass, with each box representing a habitat × organism treatment combination. Each glass contained five individuals. (B) Layout of experiment two. Circles represent single glasses, with each box representing a habitat × organism treatment combination. Each glass contained five individuals of each species.

The treatments in the experiment were the two different sources of water that we collected (MRSM and WNC). Each species was reared in water from both habitats, with five replicates in each treatment (Fig. 1). We kept animals in 20 ml of water in small glass containers (hereafter “glass”).

We collected invertebrates for the experiment from the WNC or MRSM sediment and pore water. After an invertebrate was found using a dissection microscope, a glass transfer pipette was used to place it into a replicate, with five invertebrates in each glass for a starting density of one individual per four ml. We chose this density so that animals would not be crowded at the beginning of the experiment, allowing for potential population growth. *Heterocypris* sp. A were found in the sediment from the MRSM, and *Diacyclops* sp. A were found in the WNC sediment. We did not acclimate the field-collected organisms to lab conditions, while *D. pulex* were ordered from Carolina Biological Supply Company (Burlington, NC).

Every day each glass received five drops of brewer’s yeast mixed with water, which was the food for the invertebrates. The final concentration of yeast added to the glasses was 4 * 10^4^ cells per ml, though we did not measure the decrease in concentration as the animals fed during a day. Although yeast is not an optimal food for any of our study species, it is an accepted food source for all of these groups (Maguire, 1960; Bouchnak & Steinberg, 2010; Miah et al., 2013; Magouz et al., 2021). To maintain the cultures, half the water from the glass was removed with a transfer pipette every 3 days, making sure to not suck up any invertebrates. After that, we used a pipette to refill the glass with clean, bag-filtered water from the correct habitat. Each day, the invertebrates were counted under the microscope. We considered an invertebrate dead if it did not make any movements for 2 min. Replicates were kept at room temperature (20 °C) and under ambient light conditions. Although this means that temperature and light were not tightly controlled, all replicates were subjected to the same conditions, allowing us to test the effect of water source (salt marsh *versus* freshwater) on population growth.

Because population sizes both increased (due to reproduction) and decreased (due to mortality) during the experiment, we could not calculate a simple rate of increase or decrease for each glass. Instead, we used the trapezoid rule to calculate the area under the population growth trajectory for each replicate, a variable called “integrated population density” (IPD) and which is measured with the units “individuals * day” (Searle et al., 2016). The formula to calculate IPD was (a + b)/2 * h, where a and b are the y-values (population size) for Days 1 and 2, and h the difference between days 1 and 2 (*i.e.*, 1 day). Using IPD allowed us to measure population growth as an integrated variable, with the glass as the level of replication in the statistical analyses. Replicates with higher values of IPD had overall higher population sizes during the experiment. When the assumption of homoscedasticity was met based on Bartlett’s test, we ran a t-test to compare IPD for each species in the MRSM water *versus* the WNC water. When homoscedasticity was not met, we used a Mann-Whitney U test to compare the IPDs from each habitat. All tests were run in R, version 3.5.3 (R Core Team, 2016) with the functions bartlett.test(), wilcoxon.test(), and aov() in base R.

### Experiment 2

In order to see how interspecific interaction would affect the responses of the animals to potentially stressful environments, we conducted an experiment of pairwise trials between species. Collecting the invertebrates and placing them into the glasses was the same as the first experiment, except that each glass had two different invertebrates: five of each species for ten total individuals per glass (Fig. 1). Feeding, water changing, and data collection mirrored the first experiment.

For each glass and day, we subtracted the number of species A from the number of species B to get a difference between the species. We then calculated the IPD of the difference for each glass based on the trapezoid rule as described above. If two species showed the same population trajectory, the expected IPD of differences over the course of the experiment would be zero. Therefore, to check for differences from zero, we calculated the 95% confidence interval on the mean of each treatment. If the CIs did not overlap with zero, the test indicated significant differences in IPD between the species within a habitat. The tests were conducted separately for each habitat.

## Results

### Experiment 1

For the first round of experiments, we tested how each invertebrate (*Daphnia pulex, Heterocypris* sp. A, and *Diacyclops* sp. A) would fare in water taken from the Kalamazoo River (WNC) and the Maple River salt marsh (MRSM).

**Figure 2:**
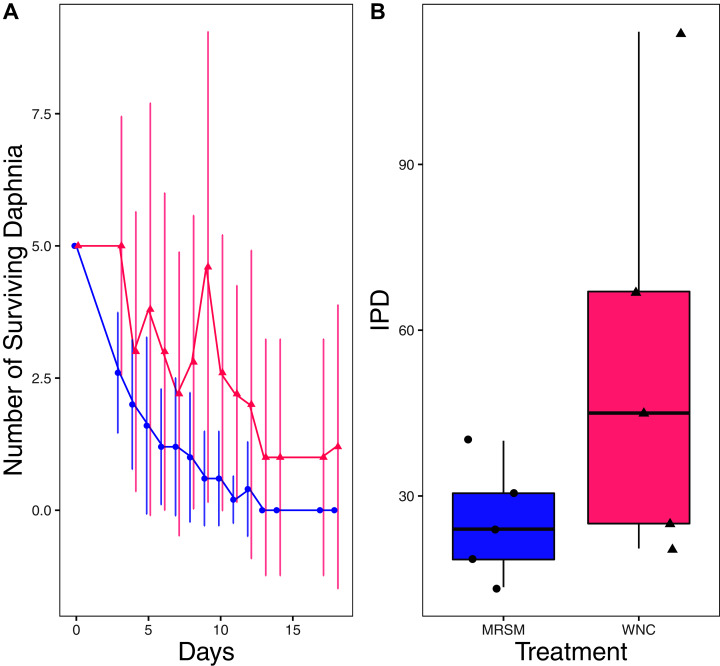
Experiment 1: *Daphnia pulex* in water from different habitats. (A) Number of *Daphnia pulex* in each treatment in the Whitehouse Nature Center (WNC; pink triangles) and the Maple River Salt Marsh (MRSM; blue circles). Dots represent treatment means, and error bars represent standard deviation. (B) Integrated population density (IPD) for *D. pulex* in each type of water. The midline on the boxplots represents the treatment medians, and dots represent individual replicates. No significant difference in IPD was found between the two different types of water.

**Figure 3:**
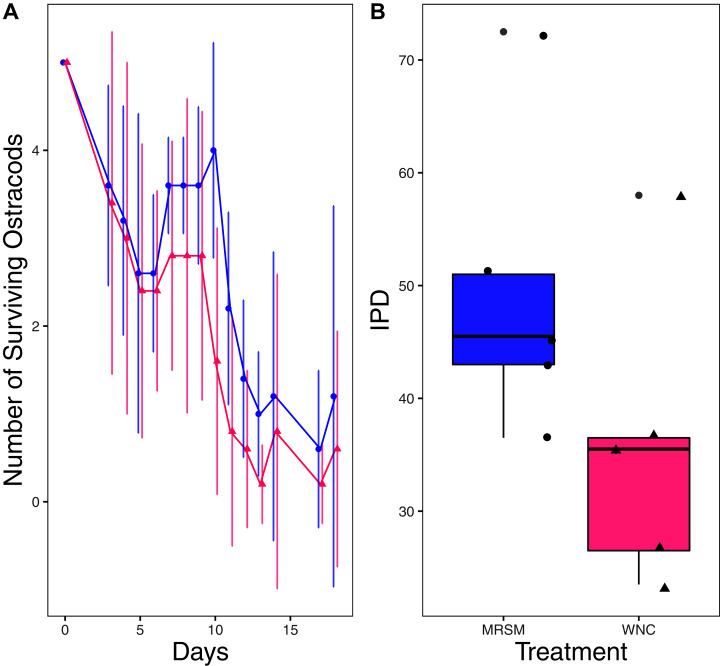
Experiment 1: *Heterocypris* sp. A in water from different habitats. (A) Number of ostracods (*Heterocypris* sp. A) in each treatment, Whitehouse Nature Center (WNC; pink triangles) and Maple River Salt Marsh (MRSM; blue circles). Dots represent treatment means, and error bars represent standard deviation. (B) Integrated population density (IPD) for ostracods in each type of water. The midline on the boxplots represents the treatment medians, and dots represent individual replicates. No significant difference in IPD was found between the two different types of water.

**Figure 4:**
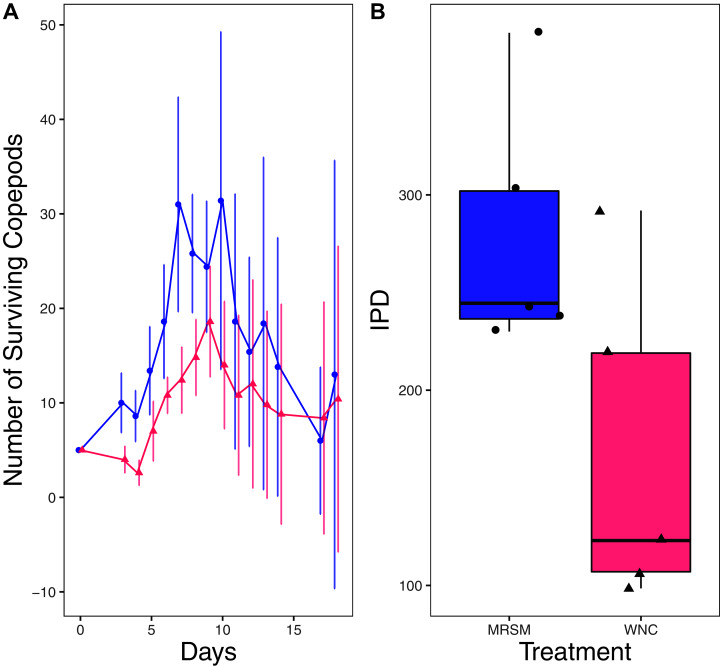
Experiment 1: *Diacyclops* sp. A in water from different habitats. (A) Number of copepods (*Diacyclops* sp. A) in each treatment, Whitehouse Nature Center (WNC; pink triangles) and the Maple River Salt Marsh (MRSM; blue circles). Dots represent treatment means, and error bars represent standard deviation. (B) Integrated population density (IPD) for copepods in each type of water. The midline on the boxplots represents the treatment medians, and dots represent individual replicates. A significant difference was found showing higher IPD in the water taken from the MRSM.

There was no significant difference when comparing integrated population density (the area under the population growth curves, measured in individuals * days) of *D. pulex* over time in the two different types of water (mean MRSM = 25.3 ± 10.37 SD, mean WNC = 54.3 ± 38.12 SD, Wilcoxon W = 5, *p* = 0.1508; Fig. 2; though note that two glasses were lost for the WNC treatment, reducing statistical power). Similarly, there was no significant difference when comparing integrated population density of *Heterocypris* sp. A over time in the two different types of water (mean MRSM = 49.7 ± 13.77 SD, mean WNC = 36 ± 13.52 SD, t = 1.5876, df = 8, *p* = 0.1510; Fig. 3). However, integrated population density of *Diacyclops* sp. A was significantly higher in the MRSM water than at the WNC (mean MRSM = 279.2 ± 64.67 SD, mean WNC = 167.9 ± 84.49 SD, t = 1.5876, df = 8, *p* = 0.0475; Fig. 4).

### Experiment 2

For the second round of experiments there were still two different sources of water being tested. However, this time two different kinds of invertebrates were paired in each replicate and population growth of both species was measured.

**Figure 5:**
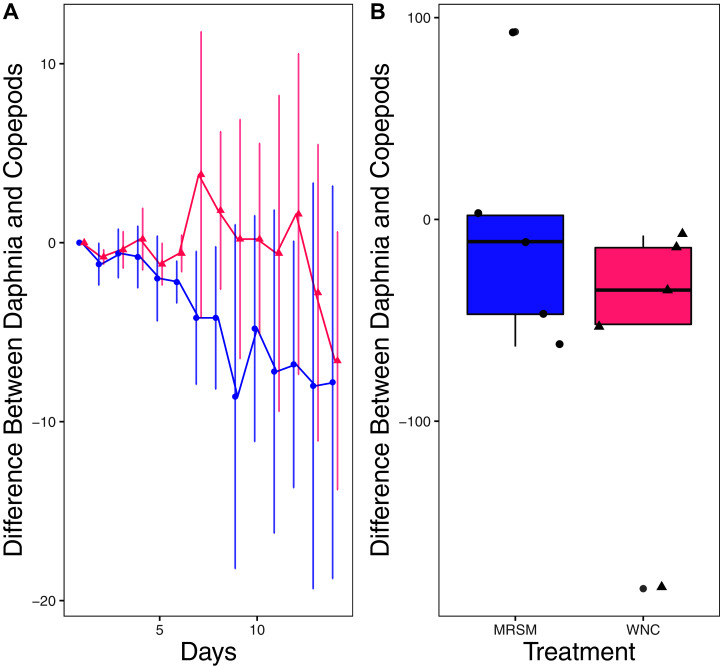
Experiment 2: *D. pulex* and *Diacyclops* sp. A in water from different habitats. (A) Difference in number of *Daphnia pulex* and the number of copepods (*Diacyclops* sp. A) in each treatment: the Whitehouse Nature Center (WNC, pink triangles) and the Maple River Salt Marsh (MRSM, blue circles). Positive values indicate more *D. pulex* than copepods. Dots represent treatment means, and error bars represent standard deviation. (B) Integrated population density (IPD) for the difference between *D. pulex* and copepods in each type of water. The midline on the boxplots represents the treatment medians, and dots represent individual replicates. The difference between the species was not different from zero in either water type.

**Figure 6:**
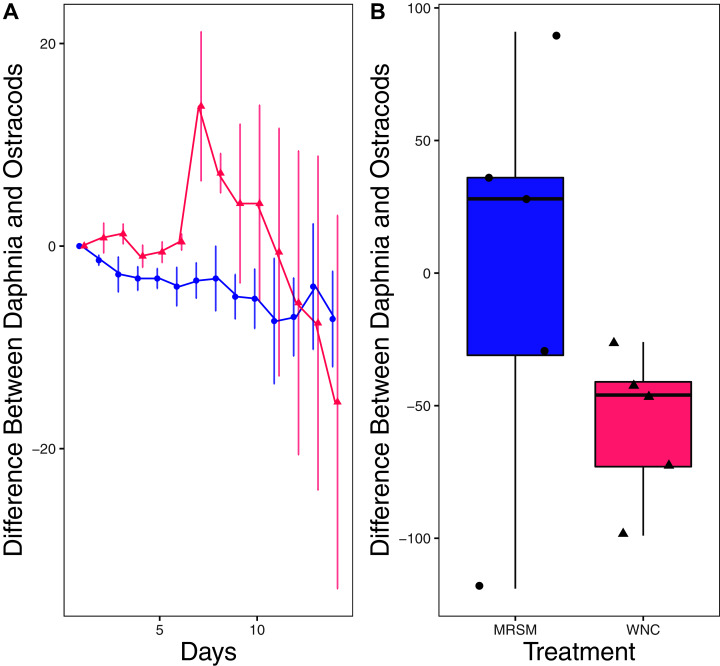
Experiment 2: *D. pulex* and *Heterocypris* sp. A in water from different habitats. (A) Difference in number of *Daphnia pulex* subtracted by the number of ostracods (*Heterocypris* sp. A) in each treatment: the Whitehouse Nature Center (WNC; pink triangles) and the Maple River Salt Marsh (MRSM; blue circles). Positive values indicate more *D. pulex* than ostracods. Dots represent treatment means, and error bars represent standard deviation. (B) Integrated population density (IPD) for the difference between *D. pulex* and ostracods in each type of water. The midline on the boxplots represents the treatment medians, and dots represent individual replicates. The difference between the species was not different from zero in the MRSM, but in the WNC ostracods had higher IPD than *D. pulex*.

**Figure 7:**
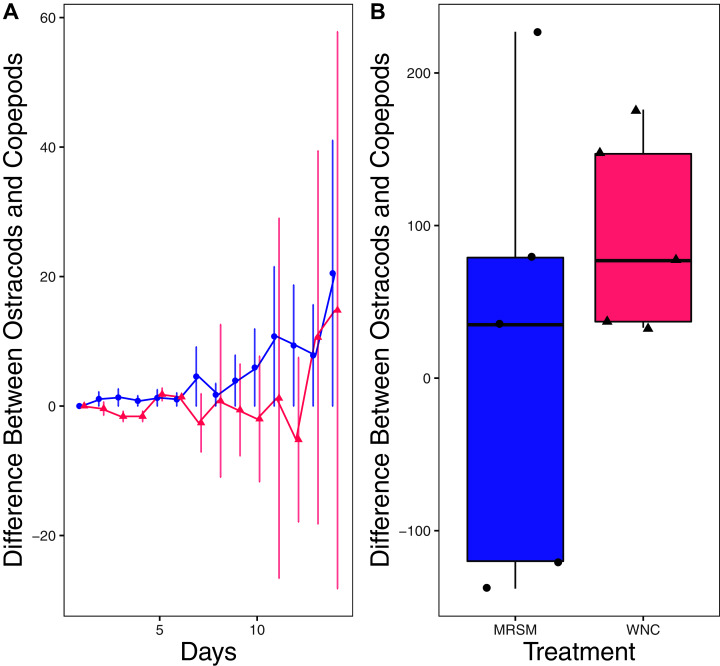
Experiment 2: *Heterocypris* sp. A and *Diacyclops* sp. A in water from different habitats. (A) Difference in number of ostracods (*Heterocypris* sp. A) subtracted by the number of copepods (*Diacyclops* sp. A) in each treatment: the Whitehouse Nature Center (WNC; pink triangles) and the Maple River Salt Marsh (MRSM; blue circles). Positive values indicate more ostracods than copepods. Dots represent treatment means, and error bars represent standard deviation. (B) Integrated population density (IPD) for the difference between copepods and ostracods in each type of water. The midline on the boxplots represents the treatment medians, and dots represent individual replicates. The difference between the species was not different from zero in the MRSM, but in the WNC ostracods had higher IPD than copepods.

There was no significant difference in integrated population density when comparing *D. pulex* and *Diacyclops* sp. A, in either water from the WNC or from the MRSM (mean difference MRSM −5.2 ± 47.715 95% CI, mean difference WNC −55.2 ± 58.473 95% CI; Fig. 5). Similarly, there was no significant difference in integrated population density when comparing *D. pulex* and *Heterocypris* sp. A in the water collected from the MRSM (mean difference MRSM 1.0 ± 62.57 95% CI). However, there was a difference in integrated population density comparing the same species in water collected from the WNC, with *Heterocypris* sp. A having the advantage (mean difference WNC −57.0 ± 22.72 95% CI; Fig. 6). There was no significant difference in integrated population density when comparing *Heterocypris* sp. A and *Diacyclops* sp. A in water collected from the MRSM (mean difference MRSM 16.6 ± 118.3 95% CI), but there was a difference in integrated population density comparing the same species in water collected from the WNC with *Heterocypris* sp. A again having the advantage (mean difference WNC 94.0 ± 50.8 95% CI; Fig. 7).

## Discussion

### Single-species responses to water source

Most organisms prefer their environment of origin, but all species also need the ability to cope with stressful conditions. Abiotic conditions such as temperature, sunlight, precipitation, and pollution all can harm and kill organisms when at suboptimal levels. Keeping this in mind, one may expect sensitive organisms to perish if placed in an unfamiliar and stressful habitat. However, this was not what we found in the first experiment. When each species was tested singly in salt or freshwater, we saw that *Diacyclops* sp. A had larger population sizes in the salt marsh environment. However, *D. pulex* and *Heterocypris* sp. A showed no difference between environments. This does not support our predictions: we expected the *D. pulex* and *Diacyclops* sp. A to have a higher integrated population density (IPD) in the freshwater from the WNC than the salt marsh, and for the *Heterocypris* sp. A to have a higher IPD in water from the salt marsh. This pattern would have shown that organisms thrive in their ‘home’ environments. Since this was not the case, no organism in in this experiment exhibited a pattern of local adaptation.

The pattern found in our experiment raises the question of why *Diacyclops* sp. A had a larger population in their ‘away’ environment, as well as why *D. pulex* and *Heterocypris* sp. A showed no differences. As a group, copepods are found in a multitude of environments, lakes, salt marshes, shorelines and many more habitats (Coull et al., 1979; Lee, Remfert & Gelembiuk, 2003; Barrera-Moreno et al., 2015). They also have a history of invading freshwater environments from a saltier home environment (Lee, 1999; Lee, Remfert & Gelembiuk, 2003) due to their ability to adapt to their local environment’s salinity. This general ability to acclimate and adapt to local salinity regimes, together with the fact that the salinities of the MRSM and the WNC are relatively similar (2.5 ppt and 0.3 ppt during our experiment, respectively) means that although we were surprised that the copepods in our experiment performed well in the salt marsh water, it is not totally out of the blue given their physiology.

In future research, a focus on species-level identification of the *Diacyclops* sp. A could provide important information about the physiological tolerances of the species. Since our attempts to identify the species using DNA barcoding were unsuccessful, a detailed understanding of the salinity tolerance in this particular species is limited. While we are still unsure exactly what species of copepod were used in our experiment, it does not change the overall results: copepod population growth under lab conditions did not correspond to its field distribution, as discussed below.

Ostracods as a group also show great ability to adapt to an environment’s salinity (Gandolfi et al., 2001). That being said, we predicted that the *Heterocypris* sp. A we collected would have done better in the water they were collected in, as they did not have to acclimate to a new environment. Since they performed similarly in both habitats, it raises the question of why. As with the copepods, the precise answer depends on a precise species identification. Ostracods have been shown to exhibit different osmoregulatory habits depending on the salinity of the water that they live in, where eight ppt appears to be a critical threshold (Aladin & Potts, 1996). The salinities of the water in both the MRSM and WNC were below that eight ppt threshold, potentially making it easier for the ostracods in our experiment to transition to the WNC water. However, it is interesting to note that Ganning (1971) found that *H. salina* preferred six ppt over lower salinities. It is possible that our species is not *H. salina*, or that different populations (in Michigan *versus* in the Baltic) have different optimal salinities.

*Daphnia pulex* did not exhibit a preference for either water: they survived equally well in both habitats. While they were not collected from either the WNC or the MRSM, *Daphnia* are found in environments with low salinity. This led us to believe that they would not be able to handle the higher salinity in the MRSM, and we expected *D. pulex* to have a higher IPD in water from the WNC. The fact that our results were not significant could be because two of the glasses that the experiment was taking place in were knocked over, reducing statistical power in this experiment. However, *D. pulex* has been shown to have intraspecific variation (across clones) in salinity tolerance (Weider & Hebert, 1987; Liu & Steiner, 2017). Our experiment was not explicitly testing differences in clonal lines, but this may have contributed to the survival of some *Daphnia* in the MRSM water.

### Interspecific interactions under different salinity conditions

In experiment two, we reared pairs of species in different salinity conditions. This allowed us to move beyond abiotic stressors and see how outcomes changed with interspecific interactions. When comparing *D. pulex* and *Diacyclops* sp. A, there was no significant difference in integrated population density for either test environment. These results are not what we expected: since *Diacyclops* sp. A is native to the water taken from the Kalamazoo River, we expected them to outperform the *D. pulex* in that set of tests. *Diacyclops* sp. A also survived extremely well in the water taken from the salt marsh in experiment one, leading us to believe that they would outperform the *D. pulex* in water from the MRSM.

Something that could have skewed the number of survivors for all of the organisms is the appearance of an unknown protozoan in many but not all of the glasses filled with water taken from the MRSM. The protozoans very quickly reached high densities in the test environments and killed the crustaceans, probably through a reduction of available oxygen in the replicate. For unknown reasons this only occurred in the second experiment.

When comparing *D. pulex* and *Heterocypris* sp. A there was no significant difference in integrated population density in the water taken from the salt marsh. However, in water taken from the Kalamazoo River it was found that *Heterocypris* sp. A did significantly better than *Daphnia*. This is half of what we had expected: our prediction was that *Heterocypris* sp. A would do better in both environments when compared to *D. pulex*. While neither species showed a difference in survivorship between environments in experiment one, we thought that ostracods’ general ability to acclimate and adapt to different salinities (Gandolfi et al., 2001) would give *Heterocypris* sp. A the edge to outperform their counterpart in both environments. Additionally, the *D. pulex* individuals we used were not native to Michigan.

When comparing *Heterocypris* sp. A and *Diacyclops* sp. A we found no significant difference when comparing integrated population density in the water collected from the salt marsh. In water taken from the Kalamazoo River, *Heterocypris* sp. A did significantly better than *Diacyclops* sp. A. When comparing the results from both experiments for *Diacyclops* sp. A, a confusing picture is painted. When alone in experiment one, *Diacyclops* sp. A did significantly better in water taken from the salt marsh than the WNC, yet in experiment two they did not outperform either the *D. pulex* or *Heterocypris* sp. A in the water taken from the salt marsh. Additionally, *Heterocypris* sp. A did not perform better in either water in experiment one. It is possible that *Heterocypris* sp. A was consuming *D. pulex* or *Diacyclops* sp. A, as some species of ostracods eat other zooplankton (Campbell, 1995), or that its advantage was due to larger starting biomass (as we did not standardize biomass between species in experiment 2). However, if these factors influenced the results, it did not happen equally in both water types. Another possibility is that the presence of another species is facilitating the population growth of the ostracods, which was shown in *Heterocypris incongruens* when grown with a cladoceran (Fernandez et al., 2016).

### Implications for species’ distributions in the field

The results of our experiments were inconsistent with the field distributions of both the ostracods and copepods (though note that we have not conducted a broad regional survey of the species). When samples were taken at the MRSM in previous studies from our group and analyzed with both morphological and molecular tools (Cahill et al., 2021), *Heterocypris* sp. A were mostly found at the site of the seep and sites 20 and 40 m away from the seep, but then dropped off with increasing distance. Our experiments here showed that these *Heterocypris* sp. A perform equally well in water from the two habitats, so it is unclear why the ostracods are localized at the salt seep in the field. One reason might be that when observed at the MRSM, the ostracods were only found in the top layer of loose sediment in the water (as has been seen in other species, *e.g.*, Ozawa, 2004). More vegetation grows farther from the seep, reducing the amount of loose sediment in the habitat. If the ostracods prefer to live in loose sediment, they may not be able to live in areas with vegetation. At the MRSM, this would localize them to the seep itself (Lincoln et al., 2020). Other biotic factors, such as microbial activity, might be related to the distribution of ostracods at the seep as well.

Another unexpected result was the tolerance of *Diacyclops* sp. A to the salinity of the salt marsh. According to experiment one, copepods were most able to cope with a new stressor. They did significantly better in MRSM water, which had a higher salinity than they were used to. However, they are not found in the marsh, at least not consistently: in 3 years of sampling in the MRSM, we did not observe any copepods, either using microscope-based identifications or molecular metabarcoding (Cahill et al., 2021). It is possible that they are not naturally found at the salt marsh because the level of salinity it reaches could be past the livable range of the species. Although the copepods in the experiment performed well at 2.5 ppt over the short term, salinities in the marsh can reach at least as high as five ppt (Cahill et al., 2021), and we did not measure reproduction or the effect of high salinity on early life stages of the copepods. However, other species of *Diacyclops* have been found living in caves at and above the maximum salinity measured at the MRSM (Gottstein et al., 2007).

The water level at the seep is variable and can be up to two feet deep (Lincoln et al., 2020) but is highly dependent on that season’s rain. It is therefore possible for the marsh to dry out, and in fact, in July of 2018 the seep had no water in it (A.E. Cahill, 2018, pers. obs.). Although some species of copepods have dormant stages in their life cycles, not all do (Dahms, 1995). Our inability to identify the copepods to the species level precludes us from knowing if these copepods have such a resting stage. If they do not, this might explain their inability to persist in the variable water level of the MRSM.

Another possibility to explain the lack of copepods in the MRSM is dispersal limitation. Copepods can be introduced into new locations from bodies of water that are newly connected or *via* human interference (Albaina et al., 2016). Since the MRSM is dependent on rainfall and groundwater and is not connected to another body of water, copepods’ natural way of introduction is unlikely, though note that copepods can be introduced *via* wind or waterfowl as well (Cáceres & Soluk, 2002; Frisch, Green & Figuerola, 2007). While we do not know if copepods have ever been introduced at this site, we now have a better understanding of what might happen if a species of copepod were to make its way into the Maple River salt seep. Based upon our findings, the copepods would probably not be hindered by the salinity of this environment, but would be unlikely to outcompete the current inhabitants.

Although our study focuses on naturally occurring inland salt marshes, our results have implications for salinization in the region as well. Many places across the United States of America use large volumes of road salt to clear ice and snow from roads and sidewalks. While this practice is cheap and effective, it has led to a major problem: the salty runoff is ending up in many local freshwater environments (Dugan et al., 2017; Kaushal et al., 2021). Our results show that some freshwater invertebrates may be more sensitive to salinization than others, and concur with previous studies finding that some freshwater copepods have been able to survive some amounts of road salt being added to their environment (Dugan et al., 2017).

Due to the controlled nature of these lab experiments, to obtain better results many factors were left out that could affect an organism’s ability to outperform another under more natural conditions. These factors could include other organisms, oxygen levels, or food availability, which could change which organism outcompetes the other. In addition, the water for these experiments was collected at the beginning of the experiment and left in the lab after filtering. This means that although we know salinity did not change measurably during the experiment, other chemical or microbial conditions may have. Since all organisms were reared in the same water, this is unlikely to affect the differences we saw between organisms.

Additionally, we did not measure the mortality or reproduction of each species in our experiments, instead relying on IPD as a metric to combine the two parameters. We did not measure mortality because the microcrustacean bodies tended to decompose quickly after death. However, we note that the three species may have different scopes for reproduction. In particular, *Daphnia* and *Heterocypris* can both reproduce parthenogenetically, while the copepods cannot. We did not explicitly test for clonal lines in the species that have the ability to reproduce parthenogenetically, so we cannot say that the diversity in the gene pool would match what might be found in the wild; it is also possible that the field-collected species in fact contain cryptic species, though we think this is unlikely due to the very small size of the collection site. We did not measure sex ratio in the copepods, nor did we look for the presence of eggs in any of the species, when setting up the experiment (though we note that all species did increase in population at least some of the time). Collecting mortality *versus* reproduction data separately would give a more thorough picture of population growth in these habitats. *Daphnia pulex* were purchased from lab cultures, while the ostracods and copepods were field-collected and not lab-acclimated; this also may have influenced the species’ ability to reproduce in the experimental conditions.

## Conclusions

In experiment one we thought that the selected invertebrates would have greater numbers in their natural habitat. This was not the case. The copepods had a larger population in a foreign environment and the *Daphnia* and ostracods did not do better or worse in either environment. In experiment two we expected the invertebrate which came from the water that the test took place in to outperform the other, and for the *Daphnia* to be outperformed in both waters by both invertebrates. We did not find this pattern; although ostracods outperformed both *D. pulex* and copepods in the WNC water, this was not their natural habitat. In all other matchups, no matter the water or invertebrate pairing, neither species outperformed the other. These results contribute to a better understanding of the relationships between *Heterocypris* sp. A, *Diacyclops* sp. A, and *D. pulex* in varying salinities and give rise to several additional research questions, which may help manage or conserve the rare habitat of the inland salt marsh. With rare environments around the globe shrinking rapidly it is important that more information is found while these environments still exist.

## Supplemental Information

10.7717/peerj.12378/supp-1 Supplemental Information 1 Raw dataset for Experiment 1. Each datapoint represents the number of individuals of a particular invertebrate alive on a given day, in a given experimental replicate and treatment.

10.7717/peerj.12378/supp-2 Supplemental Information 2 Raw data from experiment 2. Each datapoint represents the number of individuals of a particular invertebrate alive on a given day, in a given experimental replicate and treatment. The information is given for each of two species in each replicate.

## Additional Information and Declarations

### Field Study Permissions

The following information was supplied relating to field study approvals (*i.e*., approving body and any reference numbers): Access and collection at the Maple River salt marsh site was approved by the Michigan Department of Natural Resources, permit MR-01-19.

### Data Availability

The following information was supplied regarding data availability: The raw data is available in the Supplemental Files.
